# Viruses Infecting a Freshwater Filamentous Cyanobacterium (*Nostoc* sp.) Encode a Functional CRISPR Array and a Proteobacterial DNA Polymerase B

**DOI:** 10.1128/mBio.00667-16

**Published:** 2016-06-14

**Authors:** Caroline Chénard, Jennifer F. Wirth, Curtis A. Suttle

**Affiliations:** a Department of Earth Ocean and Atmospheric Sciences, University of British Columbia, Vancouver, British Columbia, Canada; b Departments of Botany and Microbiology & Immunology, University of British Columbia, Vancouver, British Columbia, Canada; c Institute for the Oceans and Fisheries, University of British Columbia, Vancouver, British Columbia, Canada; d Canadian Institute for Advanced Research, Toronto, Ontario, Canada

## Abstract

Here we present the first genomic characterization of viruses infecting *Nostoc*, a genus of ecologically important cyanobacteria that are widespread in freshwater. Cyanophages A-1 and N-1 were isolated in the 1970s and infect *Nostoc* sp. strain PCC 7210 but remained genomically uncharacterized. Their 68,304- and 64,960-bp genomes are strikingly different from those of other sequenced cyanophages. Many putative genes that code for proteins with known functions are similar to those found in filamentous cyanobacteria, showing a long evolutionary history in their host. Cyanophage N-1 encodes a CRISPR array that is transcribed during infection and is similar to the DR5 family of CRISPRs commonly found in cyanobacteria. The presence of a host-related CRISPR array in a cyanophage suggests that the phage can transfer the CRISPR among related cyanobacteria and thereby provide resistance to infection with competing phages. Both viruses also encode a distinct DNA polymerase B that is closely related to those found in plasmids of *Cyanothece* sp. strain PCC 7424, *Nostoc* sp. strain PCC 7120, and *Anabaena variabilis* ATCC 29413. These polymerases form a distinct evolutionary group that is more closely related to DNA polymerases of proteobacteria than to those of other viruses. This suggests that the polymerase was acquired from a proteobacterium by an ancestral virus and transferred to the cyanobacterial plasmid. Many other open reading frames are similar to a prophage-like element in the genome of *Nostoc* sp. strain PCC 7524. The *Nostoc* cyanophages reveal a history of gene transfers between filamentous cyanobacteria and their viruses that have helped to forge the evolutionary trajectory of this previously unrecognized group of phages.

## IMPORTANCE

Filamentous cyanobacteria belonging to the genus *Nostoc* are widespread and ecologically important in freshwater, yet little is known about the genomic content of their viruses. Here we report the first genomic analysis of cyanophages infecting filamentous freshwater cyanobacteria, revealing that their gene content is unlike that of other cyanophages. In addition to sharing many gene homologues with freshwater cyanobacteria, cyanophage N-1 encodes a CRISPR array and expresses it upon infection. Also, both viruses contain a DNA polymerase B-encoding gene with high similarity to genes found in proteobacterial plasmids of filamentous cyanobacteria. The observation that phages can acquire CRISPRs from their hosts suggests that phages can also move them among hosts, thereby conferring resistance to competing phages. The presence in these cyanophages of CRISPR and DNA polymerase B sequences, as well as a suite of other host-related genes, illustrates the long and complex evolutionary history of these viruses and their hosts.

## INTRODUCTION

Filamentous cyanobacteria of the genera *Nostoc* and *Anabaena* are abundant and active members of aquatic and terrestrial microbial communities. They occur in habitats ranging from the bottom of ice-covered polar lakes (1) and hypertrophic coastal lagoons (2) to rice paddy soils (3) and rock pool communities in karst regions (4). They fix nitrogen in nutrient-poor environments and can form symbiotic associations with a wide range of plants and fungi (4). Despite the ecological importance of these cyanobacteria, the genomic composition of the viruses infecting them remains largely unexplored.

Bacteria and their viruses (phages) have a shared evolutionary history stretching for billions of years that has led to a myriad of adaptations for cells to avoid infection and countermeasures for phage to escape these defenses (5). One of these defense mechanisms involves the clustered regularly interspaced short palindromic repeats (CRISPRs) and the CRISPR-associated (*cas*) genes (6, 7) (i.e., the CRISPR-Cas system). Evidence of this adaptive immune system is found in almost all archaeal genomes and in about 40% of bacterial genomes (8). The CRISPR array consists of a series of noncontiguous direct repeats (DRs) 20 to 50 bp long that are separated by variable sequences (spacers) usually derived from viruses and plasmids (6, 7, 9). A leader region that is an AT sequence of up to 550 bp directly adjoins the first regions. In some cases, *cas* genes are found upstream or downstream of CRISPR arrays. The CRISPR-Cas system provides immunity to cells by recognizing and cleaving incoming foreign genetic material with sequence similarity to the spacers (6–8, 10). Many types of CRISPRs have been recognized on the basis of the sequence similarity of the repeats (11). The distribution of closely related CRISPR/Cas systems in phylogenetically distant organisms suggests exchange by horizontal gene transfer (12). CRISPR/Cas systems are rare in plasmids and prophages; however, it has been suggested that they could mediate the exchange of CRISPRs among organisms (13–15).

Phages infecting *Nostoc* spp. were among the first cyanophages to be studied extensively (16), including host range, life cycle, and host interaction (17–19). For example, cyanophage N-1 was the first shown to reduce host photosynthesis upon infection (20). Despite the extensive work on the viruses and the effects of infection on the hosts, the viruses have remained uncharacterized genomically. In this study, we analyzed the genome sequences of viruses infecting the freshwater filamentous cyanobacterium *Nostoc* sp. strain PCC 7120. Our results demonstrate that cyanophages A-1 (21) and N-1 (17, 22) are distantly related to other genetically characterized cyanophages. Both viruses contain a distinct DNA polymerase B (Pol B) that is closely related to those found in plasmids of their cyanobacterial hosts. In addition, cyanophage N-1 has a CRISPR array that is similar to those found in cyanobacteria, suggesting that cyanophages play a role in exchanging CRISPRs among cyanobacteria.

## RESULTS AND DISCUSSION

The sequencing of cyanophages A-1 and N-1, which infect *Nostoc* sp. strain PCC 7120, revealed a previously unknown lineage of myoviruses. Few of the predicted coding genes were similar to those found in other cyanophages, while the DNA Pol B sequences were similar to those found in a host plasmid. In addition, cyanophage N-1 contains a CRISPR array similar to those found in cyanobacteria, suggesting that phages may mediate the exchange of CRISPRS among cyanobacteria and confer resistance to competing phages.

### Genome features.

The double-stranded DNA (dsDNA) genomes of cyanophages A-1 and N-1 are 68,304 and 64,960 bp long, with G+C contents of 38.3 and 35.4%, respectively, and have circularly permuted, terminally redundant ends. Their genome lengths are about half those of many other phages that have an obvious contractile tail. For example, coliphage T4 has a genome of 168 kb (23), while other myoviruses have genomes ranging from 161 to 231 kb in length (24–29). However, some viruses with contractile tails have much smaller genomes; the classic case is bacteriophage Mu, a temperate bacteriophage of 36 kb that reproduces by transposition. In addition, there are recently characterized (30) “dwarf” myoviruses that have genomes of <50 kb and infect a diversity of proteobacteria (i.e., *Aeromonas salmonicida*, *Vibrio cholerae*, *Bdellovibrio* spp., and *Pectobacterium carotovorum*). These viruses do not have sequence similarity to A-1 and N-1.

Bioinformatic analysis of A-1 and N-1 revealed that only about a quarter of the translated open reading frames (ORFs) had similarity to protein sequences in current databases (36 of 97 for A-1 and 33 of 91 for N-1). This is similar to other freshwater cyanophages, including recently described S-EIV1, in which 85% of the translated ORFs had no database matches (31), and S-CRM01 (26), Ma-LMM01 (29), Pf-WMP4, and Pf-WMP3 (32, 33), which range from 61 to 76% similarity. In contrast, only about 20% of the ORFs in most phage isolates do not have recognizable homologues (34), while in marine cyanophages, the percentage is typically <40% (27, 35). Clearly, there is a lack of representative genomes of freshwater cyanophages.

**FIG 1:**
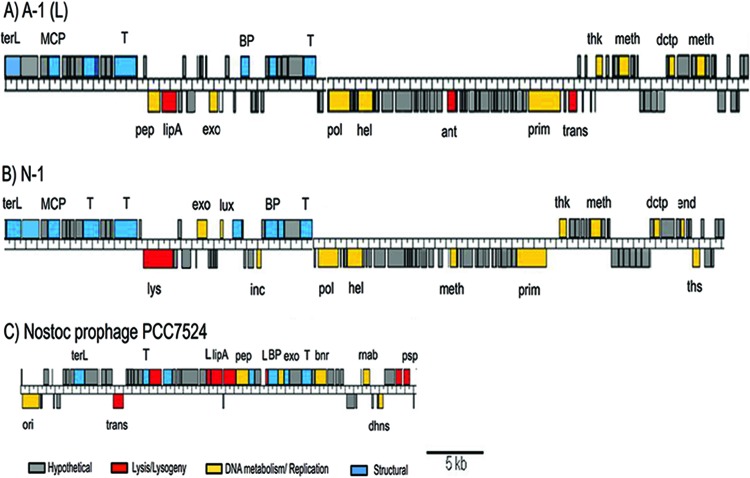
Comparative genomics of the two *Nostoc* cyanophages and the prophage-like element in the genome of *Nostoc* sp. strain PCC 7524. The genomes are presented as linear molecules for better comparison. A, cyanophage A-1; B, cyanophage N-1; C, prophage-like element in the genome of *Nostoc* sp. strain PCC 7524 (accession no. NC019684.1). Gene abbreviations and functions are as follows: ant, antirepressor; BP, baseplate; bnr, bnr/Asp-box repeat protein; dctp, dCTP deaminase; dhns, dihydroxynaphthoate synthase; exo, exonuclease; hel, helicase; incl, viral A inclusion factor; L, lysozyme; lipA, lipoprotein A; luX, LuxR transcription factor, lyz, lysozyme; MCP, major capsid protein; meth, methylase; ori, origin of replication; pep, peptidase; psp, phage shock protein; pol, DNA Pol B; prim, primase; T, tail; terL, terminase large subunit; thk, thymidylate kinase; ths, thymidylate synthase; trans, transposase.

Translated ORFs with significant hits to proteins of known function were associated with DNA replication, DNA metabolism and repair, and structural components (Fig. 1; see Tables S1 and S2 in the supplemental material). There were also some putative genes consistent with a temperate lifestyle, such as a transposase and two phage antirepressors, in cyanophage A-1. Although no obvious repressor genes were identified, a plausible candidate is a putative LuxR-encoding gene that was found in both genomes. However, no evidence of genes encoding an integrase or partitioning proteins was found.

Although there was high similarity between the *Nostoc* cyanophages, they had little similarity to cyanophages infecting other cyanobacteria. Genomic studies of 26 T4-like phages, including 16 marine cyanophages, identified 38 T4-like core genes coding primarily for structural and DNA replication proteins (35). In contrast, only six T4-like core genes have significant similarity to ORFs in the *Nostoc* cyanophages (Table 1), and those genes are associated with replication and DNA modification. In addition, none of the 25 genes found exclusively within marine cyanomyoviruses were present in the *Nostoc* viruses.

**TABLE 1:** Predicted ORFs in cyanophages A-1 and N-1 with similarity to T4-like genes

T4 core gene product	CyanophageA-1		Cyanophage N-1	
	E value	Phage hit	E value	Phage hit
gp5 baseplate hub + tail lysozyme	4.00e^−5^	Phage RB49	4.00e^−5^	Cyanophage S-PM2
gp17 terminase DNA-packaging enzyme large subunit	4.00e^−2^	Cyanophage S-PM2	4.00e^−2^	Cyanophage S-PM2
Gp41 DNA primase-helicase	2.00e^−13^	Cyanophage S-PM2	1.00e^−11^	Cyanophage S-RSM4
gp43 DNA Pol B	4.00e^−5^	Phage Aeh1	9.00e^−6^	Phage Aeh1
Thymidylate synthase	1.00e^−35^	Cyanophage S-CRM01	9.00e^−32^	Cyanophage P-SSM4

Both genomes were analyzed for regulatory elements and motifs such as tRNA genes, promoter motifs, and transcriptional terminators. Unlike viruses infecting marine cyanobacteria, but not unusual for myoviruses with genomes smaller than 70 kb, tRNA genes were not identified in cyanophages A-1 and N-1. A recent study found that 7 of 10 small myoviruses did not contain tRNAs (30). In addition, the presence or absence of tRNAs might influence host range, as myoviruses that infect only *Prochlorococcus* spp. tend to code for few, if any, tRNAs, whereas those infecting *Prochlorococcus* spp. and *Synechococcus* spp. or just *Synechococcus* spp., code for more tRNAs (36). In contrast, early studies described cyanophages A-1 and N-1 as being able to infect both *Anabaena* and *Nostoc* spp. (19); however, it has been suggested that the reported host range might reflect errors in taxonomy (16, 37).

**FIG 2:**
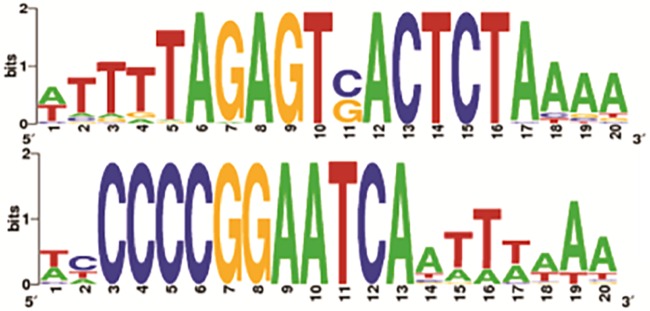
Sequence logos of the promoter motifs predicted from alignments of the 5′ upstream regions (top) and putative promoter for cyanophage A-1 showing a sequence logo created from an alignment of 43 sequences (bottom). The putative promoter for cyanophage N1 shows a sequence logo created from an alignment of 21 sequences. The heights of the letters are proportional to the levels of sequence conservation of the nucleotides at the respective positions.

Putative promoter motifs in the *Nostoc* cyanophage genomes were identified with PHIRE (38), although the sequences differed (Fig. 2). For A-1, 43 motifs were identified that contained a consensus sequence for a putative promoter motif consisting of two highly conserved regions separated by 1 bp (Fig. 2). For N-1, 21 motifs were identified that included a highly conserved region of 11 bp in the repeat motif. Consistent with functioning as promoters, the motifs were typically located between ORFs. However, the motifs were asymmetric and not consistently in the same direction as the flanking genes, which suggests that they may not function as promoters. An alternative possibility is that the motifs represent stoperators that bind a regulatory protein such as a phage repressor, as has been shown for mycobacteriophages (39).

### Presence of a distinct DNA Pol B.

**FIG 3:**
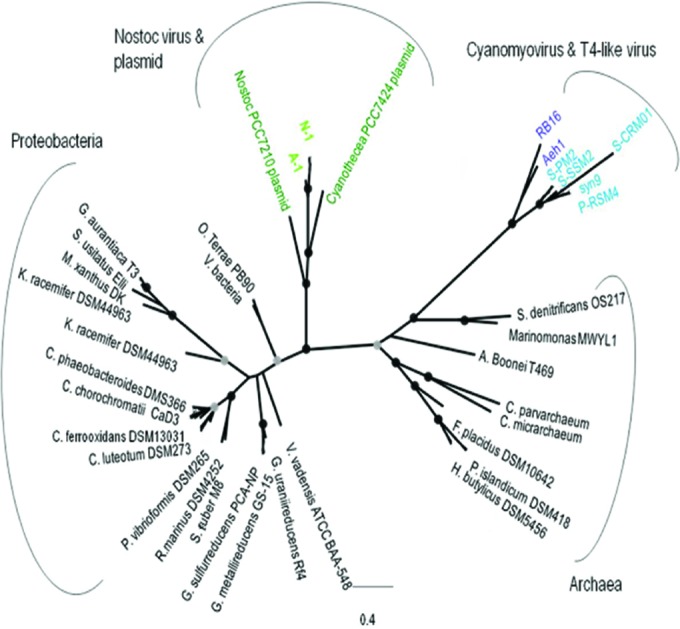
Unrooted ML phylogenetic tree of DNA Pol B protein sequences found in viruses, bacteria, and archaea. Bootstrap values of 90 to 100% (black circles) and 75 to 89 % (gray circles) are shown at the nodes. The sequence names are colored as follows: black, *Bacteria* and *Archaea*; light blue, marine cyanomyoviruses; dark blue, T4-like myoviruses; light green, *Nostoc* phages; dark green, cyanobacterial plasmid. The scale bar shows the number of amino acid substitutions per site.

Sequences were found in A-1 and N-1 that putatively encode a DNA Pol B that catalyzes the polymerization of deoxyribonucleotides into a DNA strand and has many viral and cellular homologues, making it a useful phylogenetic marker. The Pol B sequences in A-1 and N-1 were most closely related to plasmids in the cyanobacteria *Cyanothece* sp. strain PCC 7424 (plasmid pP742402), *Nostoc* sp. strain PCC 7120 (plasmid pCC7120beta), and *Anabaena variabilis* ATCC 29413 (plasmid C) (Fig. 3). These viral and plasmid Pol B sequences form a distinct group that is related to proteobacterial and archaeal Pol B clades. Moreover, while Pol B is common in proteobacteria, the only representatives known in cyanobacteria are in these three plasmids.

The close phylogenetic relationship between the Pol B sequences in the phages and plasmids suggests that the gene for Pol B was transferred laterally. Moreover, the DNA Pol B in *Cyanothece* plasmid pP742402 is adjacent to a CRISPR, a region where recombination can occur and thus be promiscuous to gene exchange. The DNA polymerases in A-1, N-1, and cyanobacterial plasmids have an ancestor in common with proteobacteria, implying that the transfer of DNA to the cyanobacterial plasmids may have been phage mediated. Recent genomic analysis of other cyanophages reveals the presence of interesting polymerases. For instance, viruses infecting the marine cyanobacterium *Acaryochloris marina* contain a Pol A that appears to be related to eukaryotic polymerases (40). In addition, a polar cyanophage (S-EIV1) recently isolated from polar inland waters contains a Pol A sequence that is phylogenetically divergent from those found in other cyanophages (31).

### Phylogeny of the terminase large subunit.

**FIG 4:**
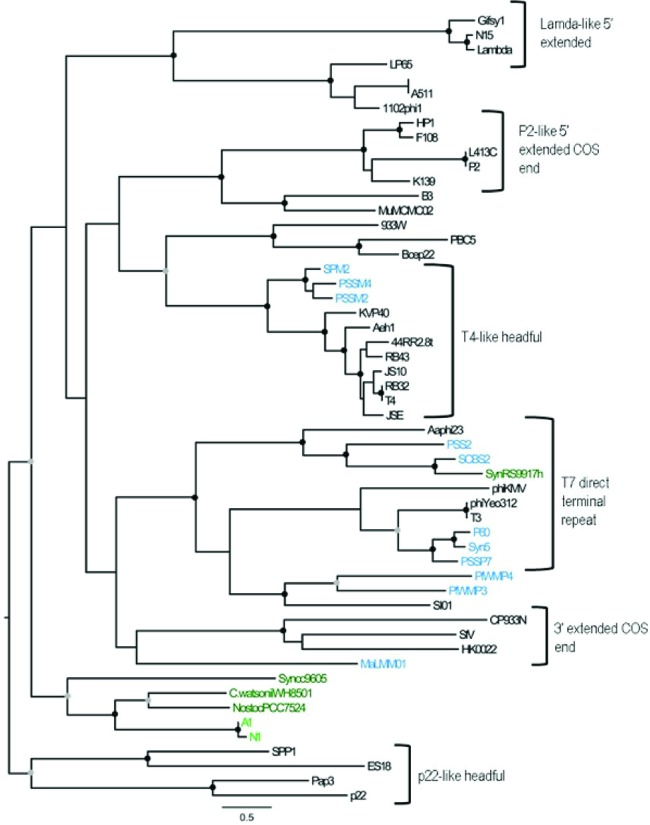
Phylogenetic relationship of terminase large subunit protein sequences from phages. A ML tree is shown with bootstrap values of 90 to 100% (black circles) and 75 to 89 % (gray circles) shown at the nodes. The sequence names are colored as follows: light green, *Nostoc* cyanophages; dark green, cyanobacteria; light blue, other cyanophages; black, other viruses.

Further evidence of the evolutionary divergence of A-1 and N-1 from other cyanophages is provided by the gene *terL*, which encodes the terminase large subunit, a protein involved in DNA packaging in dsDNA phages. Phylogenetic analysis reveals that the translated *terL* sequences from A-1 and N-1 cluster separately from those in other viruses (Fig. 4) and branch most closely with those in the freshwater heterocystous cyanobacterium *Nostoc* sp. strain PCC 7524 and the marine tropical and subtropical unicellular N fixer *Crocosphaera watsonii*, while marine *Synechococcus* sp. strain PCC 9605 is somewhat more distant. It has been argued that terminases are good phylogenetic markers of phage evolution and that sequences found in cyanobacteria may be remnant prophages (41). Indeed, the gene for the terminase large subunit in *C. watsonii* occurs near genes encoding a phage tail collar (NCBI reference sequence accession no. EAM53192), a transposase (NCBI reference sequence accession no. EAM53191), and a hypothetical protein (NCBI reference sequence accession no. EAM53190) that also show similarity to putative genes in the *Nostoc* cyanophages. The terminase large subunit in *Nostoc* sp. strain PCC 7524 is also part of a prophage-like element (see below). The A-1 and N-1 terminase sequences have revealed a new evolutionary group of phage terminases with similarity to prophage elements in several genera of divergent cyanobacteria, suggesting that relatives of A-1and N-1 infect a broad range of marine and freshwater hosts.

### Genetic exchange between filamentous cyanobacteria and *Nostoc* cyanophages.

Some ORFs in A-1 and N-1 exhibit high similarity to genes in cyanobacteria that code for proteins with known functions (Table 2 and 3), including purine and pyrimidine metabolism. One example is dCTP deaminase, an enzyme involved in the production of dUMP, the immediate precursor of thymidine nucleotides. Phylogenetic analysis demonstrates that dCTP deaminase sequences from the *Nostoc* cyanophages are more similar to those found in cyanobacteria, whereas the dCTP deaminase homologue in the marine cyanophage S-PM2 is more closely related to other virus sequences (see Fig. S1 in the supplemental material). Thymidylate synthase and thymidylate kinase genes in A-1and N-1 were also similar to those found in cyanobacteria. The proteins encoded by these genes likely catalyze two subsequent steps in dTTP synthesis. Thymidylate synthase is involved in the production of dTMP, while thymidylate kinase phosphorylates dTMP to dTDP. This reaction is crucial to both the *de novo* synthetic and salvage pathways for pyrimidine deoxyribonucleotides. The gene encoding thymidylate kinase is commonly found in eukaryotes and their DNA viruses and has been reported in some myoviruses with genome sizes of >200 kb (42, 43); however, to our knowledge, this is the first time a homologue of this gene in A-1 and N-1 has been reported in phages with genomes of <70 kb. Sequences with similarity to putative genes encoding DNA adenine methyltransferases and DNA cytosine methyltransferases were also found in the *Nostoc* cyanophages. In general, DNA methyltransferases mediate postreplicative methylation at a specific recognition site and protect bacterial DNA against digestion by specific restriction endonucleases, whereas unmethylated infective DNA, such as in phages, is cleaved. However, DNA methyltransferases occur in some phages and modify the viral DNA to be resistant to bacterial restriction systems*.* In general, phage DNA methyltransferases are similar to those of their hosts.

**TABLE 2:** Predicted ORFs in cyanophage A-1 with high similarity to cyanobacterial genes

ORF	Length (bp)	Strand^a^	Significant hit	Organism (accession no.)	E value	% Identity (no. of shared aa)^b^
1	1,359	F	Terminase large subunit	*Nostoc* sp. strain PCC 7524 (AFY48994.1)	2.28e^−54^	30 (129)
2	1,605	F	Hypothetical protein	*Nostoc* sp. strain PCC 7524 (AFY48995.1)	7.16e^−17^	21 (113)
9	1,521	F	Tail sheath protein	*Nostoc* sp. strain PCC 7524 (AFY49006.1)	1.14e^−69^	38 (137)
16	1,416	R	Lysozyme-like domain, rare lipoprotein A	*Anabaena variabilis* ATCC 29413	2.3e^−19^	57.7 (60)
19	738	R	Hypothetical protein	*Nostoc* sp. strain PCC 7524 (AFY49010.1)	9.39e^−11^	29 (67)
23	891	R	Exonuclease RNase T and DNA polymerase	*Cyanobacterium aponinum* PCC 10605	1.63e^−08^	27 (48)
26	813	F	Phage-related baseplate assembly protein	*Nostoc* sp. strain PCC 7524 (AFY49015.1)	1.46e^−32^	34 (91)
32	738	F	Baseplate J phage tail	*Nostoc* sp. strain PCC 7524 (AFY49018.1)	3.76e^−33^	41 (94)
34	576	F	Phage tail protein (tail_P2_I)	*Nostoc* sp. strain PCC 7524 (AFY49020.1)	5.63e^−40^	59 (77)
35	1,389	F	Hypothetical protein	*Nostoc* sp. strain PCC 7524 (AFY49021.1)	4.83e^−57^	37 (157)
36	1,140	F	Tail collar protein	*Nostoc* sp. strain PCC 7524 (AFY49022.1)	2.1e^−52^	50 (149)
39	2,016	R	DNA Pol B	*Cyanothece* sp. strain PCC 7425	7.00e^−115^	40.4 (237)
54	939	R	Putative ant AntA/AntB antirepressor	*Leptolyngbya* sp. strain PCC 7375	1.90e^−24^	45 (51)
59	618	R	DNA *N*-6-adenine-methyltransferase	*Synechocystis* sp. strain PCC 7509	1.00e^−06^	27.3 (44)
72	1,209	F	Transposase	*Nostoc* sp. strain PCC 7120	0	388 (100)
75	417	F	Hypothetical protein	*Calothrix* sp. strain PCC 7103	2.59e^−48^	58 (98)
86	600	F	dCTP deaminase/dUTPase superfamily	*Cyanothece* sp. strain PCC 7425	2.00e^−76^	67.3 (134)
89	888	F	DNA methylase N-4/N-6 domain protein	*Arthrospira maxima*	1.00e^−65^	49.4 (133)
90	432	F	Endo-DNase RusA	*Cyanothece* sp. strain PCC 7425	8.00e^−04^	28.4 (29)
92	780	R	Thymidylate synthase complementing protein	*Chlorobium phaeobacteroides* BSI	4.38e^−39^	38.4 (86)

a F, forward; R, reverse.

b aa, amino acids.

**TABLE 3:** Predicted ORFs in cyanophage N-1 with high similarity to cyanobacterial genes

ORF	Length (bp)	Strand^a^	Significant hit	Organism (accession no.)	E value	% Identity (no. of shared aa)^b^
1	1,359	F	Phage terminase, large subunit	*Nostoc* sp. strain PCC 7524	2.32e^−55^	31 (134)
2	1,602	F	Hypothetical protein	*Nostoc* sp. strain PCC 7524 (AFY48995)	3.55e^−18^	22 (106)
6	330	F	Hypothetical protein	*Nostoc* sp. strain PCC 7524 (AFY49001)	9.62e^−06^	30 (30)
9	1,521	F	Tail sheath protein	*Nostoc* sp. strain PCC 7524 (AFY49006)	3.74e^−74^	39 (138)
15	2,694	R	Lysozyme-like domain, rare lipoprotein A (RlpA)-like double psi beta barrel	*Nostoc* sp. strain PCC 7524 (AFY49014)	1.48e^−37^	28 (137)
18	849	R	Hypothetical protein	*Nostoc* sp. strain PCC 7524 (AFY49010)	9.82e^−10^	27.9 (51)
19	918	F	Exonuclease RNase T and DNA Pol III	*Thauera* sp. strain MZIT	4.14e^−04^	28.4 (38)
29	330	F	Lysozyme	*Nostoc* sp. strain PCC 7524 (AFY49017)	2.51e^−03^	24 (32)
30	1,167	F	Baseplate J phage tail protein	*Nostoc* sp. strain PCC 7524 (AFY49018)	4.18e^−68^	43 (144)
31	576	F	Phage tail protein	*Nostoc* sp. strain PCC 7524 (AFY49020)	2.32E^+00^	26.3 (45)
32	1,395	F	Phage tail fiber protein	*Nostoc* sp. strain PCC 7524 (AFY49021)	2.97e^−64^	37 (172)
33	1,155	F	Tail collar protein	*Nostoc* sp. strain PCC 7524 (AFY49022)	1.62e^−47^	33 (137)
34	1,896	R	DNA Pol B	*Cyanothece* sp. strain PCC 7424	7.0e^−118^	41.5 (243)
48	600	R	Hypothetical protein	*Nostoc punctiforme* PCC 73102	1.10e^−06^	21.4 (40)
52	633	R	C-5 cytosine-specific DNA methylase	*Nostoc punctiforme* PCC 73102	1.81e^−05^	36.2 (34)
56	240	R	ASCH domain protein	*Clostridium* phage phiMMP02	4.06e^−12^	42.3 (33)
62	627	F	Thymidylate kinase	*Lyngbya* sp. strain PCC 8106	2.45e^−23^	36 (74)
68	1,047	F	DNA-cytosine methyltransferase	*Anabaena variabilis* ATCC 29413	1.02e^−56^	33.2(127)
75	579	R	Hypothetical protein	*Thermoanaerobacter italicus* Ab9	8.67e^−03^	32.7 (33)
76	507	R	Hypothetical protein	*Calothrix* sp. strain PCC 7103	1.73e^−47^	56 (94)
77	726	R	Hypothetical protein	*Calothrix* sp. strain PCC 7103	4.94e^−06^	30.4 (78)
79	585	F	dCTP deaminase	*Synechococcus* sp. strain PCC 7335	1.45e^−69^	65.5 (131)

a F, forward; R, reverse.

b aa, amino acids.

Putative coding sequences in A-1 and N-1, as well as in the freshwater cyanophage S-CRM01 (26) and the marine cyanophage S-PM2 (24), are similar to host-like genes encoding the rare lipoprotein A (*rlpA*). Although its function is not known, *rlpA* was strongly induced during hyperosmotic stress in *Synechocystis* sp. strain PCC 6803 (44) and is upregulated as part of the general stress response of *Synechococcus* sp. strain WH8102 grown under low-phosphate conditions (45).

### *Nostoc* cyanophage-related genes were also found in the genome of *Nostoc* sp. strain PCC 7524.

Fourteen ORFs in cyanophages A-1 and N-1 that putatively encode structural proteins, terminases, lysozymes, and peptidases had high similarity to ORFs in *Nostoc* sp. strain PCC 7524 (NCBI reference sequence accession no. NC019684.1), leading to the reannotation of about 30 kb of sequence and the identification of a prophage-like element (Fig. 1C). Although lysogeny has been reported in natural *Synechococcus* communities (46, 47), few prophage-like elements have been detected in cyanobacterial genomes; for example, none were found in a dozen marine picocyanobacterial genomes (48, 49). However, evidence of lysogeny was recently found in the genomes of *Synechococcus elongatus* strains PCC 6301 and PCC 7942 (50). The lifestyles of cyanophages A-1and N-1 have been reported to be lytic, but similar prophage-like elements in *Nostoc* sp. strain PCC 7524 suggest that related phages have the potential for lysogeny. Moreover, the presence of ORFs in the host with high similarity to sequences in A-1 and N-1 indicates that genetic exchange occurs possibly via prophage integration or homologous recombination.

### Cyanophage N-1 contains a CRISPR array.

**FIG 5:**
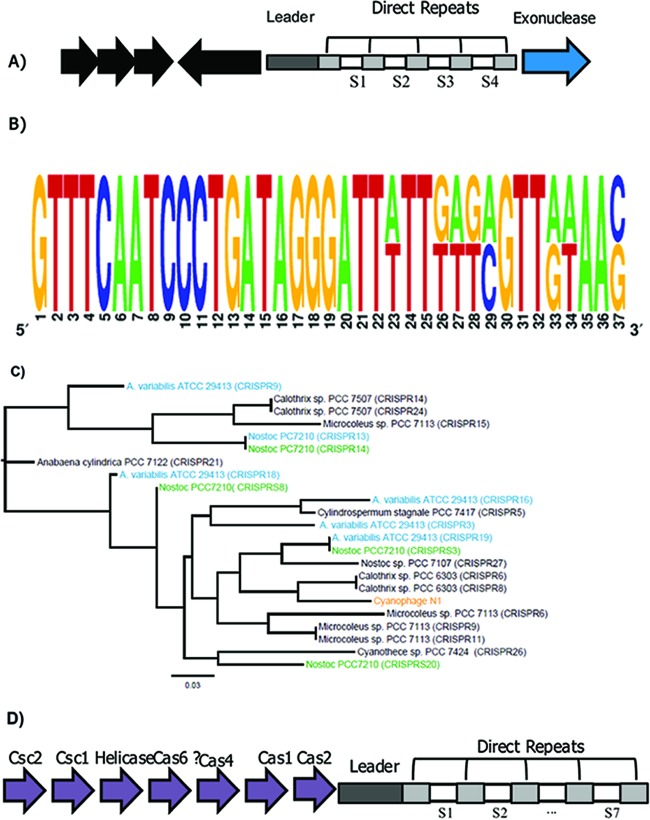
Characterization of the CRISPR in cyanophage N-1*.* (A) The CRISPR in cyanophage N-1 consists of five DRs (light gray boxes), four spacers (white boxes), and a leader sequence (dark gray box). The CRISPR array is surrounded by ORFs that putatively encode an exonuclease (blue arrows) or are hypothetical (black arrows). (B) A logo illustrating nucleotide differences in an alignment of the consensus DR from cyanophage N-1 and the consensus DR from CRISPR arrays 13 and 14 in *Nostoc* sp. strain PCC 7210. Differences are shown by placement of the letters for the nucleotides of *Nostoc* on the top and those for cyanophage N-1 on the bottom of the logo. (C) An unrooted neighbor-joining tree shows that the consensus DR in cyanophage N-1 (orange) is not most closely related to those found in its known hosts, *Nostoc* sp. strain PCC 7210 (green) and *A. variabilis* ATCC 29413 (light blue). Consensus DRs from other cyanobacteria, including those that are most closely related in *Calothrix* sp. strain PCC 6303, are shown in black. The scale bar represents 0.03 nucleotide change. (D) Schematic of CRISPR 8 in *Nostoc* sp. strain PCCC 7210. The CRISPR array consists of DRs (light gray boxes), seven spacers (white boxes), and a leader sequence (dark gray box). The CRISPR array is surrounded by CAS genes (purple arrows).

During genomic analysis of cyanophage N-1, a region of about 400 bp was identified (Fig. 5A) that comprises four spacers and five 37-bp-long DRs that are similar in structure to the DR5 family of CRISPRs commonly found in cyanobacteria (Table 4; Fig. 5B). Spacers in the N-1 CRISPR vary in length from 29 to 37 bp and from 24 to 54% in GC content (see Table S3 in the supplemental material) but did not have significant matches to other sequences in the NCBI nonredundant (nr) nucleotide database. An AT-rich sequence region (~25.6% G+C content) of approximately 120 bp, upstream of the CRISPR array, was considered the leader region. A leader region can be an AT-rich area that is upstream of the CRISPR array, as was observed in the genome of *Nostoc* phage N-1. Neighbor-joining analysis revealed that the DRs in cyanophage N-1 clustered among those in filamentous cyanobacteria (Fig. 5C) and were most similar to three sets of consensus DRs from CRISPR arrays found in the genome of *Calothrix* sp. strain PCC 7507. Overall, DRs found within a cyanobacterial genome are not necessarily most closely related to each other. For example, some CRISPRs in *Nostoc* sp. strain PCC 7210 and *A. variabilis* ATCC 29413 have repeats that are more similar to those in N-1 than to other repeats within their own genomes (Fig. 5C).

**TABLE 4:** BLAST results for the DRs from the CRISPR array present in the genome of cyanophage N-1

Cyanobacterial stain (CRISPR ID)	E value	Start	End	DR consensus sequence
*Microcoleus* sp. strain PCC 7113 (CRISPR6)	3.0e^−08^	2087079	2087333	GTCTGAATTCCATATAATCCCTATCAGGGATTGAAAC
*Microcoleus* sp. strain PCC 7113 (CRISPR11)	1.0e^−07^	3131505	3132053	GTTTAAATTCCACTTAATCCCTATCAGGGATTGAAAC
*Microcoleus* sp. strain PCC 7113 (CRISPR15)	1.0e^−07^	3859458	3860565	GTTTCAATCCCTGATAGGGATTAAGTGGAATTTAAAC
*Microcoleus* sp. strain PCC 7113 (CRISPR9)	1.0e^−07^	2916270	2917173	GTTTAAATTCCACTTAATCCCTATCAGGGATTGAAAC
*Nostoc* sp. strain PCC 7107 (CRISPR27)	1e^−07^	5817107	5818597	GTTGCAATTTCTATTAATCCCTATCAGGGATTGAAAC
*A. variabilis* ATCC 29413 (CRISPR3)	8.0e^−07^	1234556	1237182	GTTTTAATTAACAAAAATCCCTATCAGGGATTGAAAC
*Nostoc* sp. strain PCC 7210 (CRISPR13)	8.0e^−07^	3516819	3517367	GTTTCAATCCCTGATAGGGATTTTTGTTAGTTAAAAC
*Nostoc* sp. strain PCC 7210 (CRISPR14)	8.0e^−07^	3517542	3518084	GTTTCAATCCCTGATAGGGATTTTTGTTAGTTAAAAC
*A. cylindrica* PCC 7122 (CRISPR21)	4e^−07^	5001036	5003639	GTTTCAATCCCTAATAGGGATTATTTGAAATTTCAAC
*C. stagnale* PCC 7417(CRISPR5)	4e^−07^	1101502	1103134	GTTACAATTCACCCAAATCCCTATCAGGGATTGAAAC
*Calothrix* sp. strain PCC 6303 (CRISPR6)	4e^−07^	2021864	2022765	GTTCCTATAAACTAAAATCCCTATCAGGGATTGAAAC
*Calothrix* sp. strain PCC 6303 (CRISPR8)	4e^−07^	2038085	2039287	GTTCCTATAAACTAAAATCCCTATCAGGGATTGAAAC
*Calothrix* sp. strain PCC 7507 (CRISPR24)	4e^−07^	5067421	5070798	GTTTCAATCCCTGATAGGGATTTAAGTTAATTGGAAC
*Calothrix* sp. strain PCC 7507 (CRISPR14)	4e^−07^	3375025	3376306	GTTTCAATCCCTGATAGGGATTTAAGTTAATTGGAAC
*N. punctiforme* PCC 73102 (CRISPR16)	3.0e^−07^	3338172	3341197	GTTTCAATCCCTGATAGGGATTTTGATGAATTGCAAT
*Nostoc* sp. strain PCC 7210 (CRISPRS8)	3.0e^−07^	1836813	1837723	GTTTCTATTAACACAAATCCCTATCAGGGATTGAAAC
*Nostoc* sp. strain PCC 7210 (CRISPRS3)	3.0e^−07^	807452	807558	ATTGCAATTAACTAAAATCCCTATCAGGGATTGAAAC
*A. variabilis* ATCC 29413 (CRISPR19)	3.0e^−07^	5764010	5766428	ATTGCAATTAACTAAAATCCCTATCAGGGATTGAAAC
*Nostoc* sp. strain PCC 7210 (CRISPRS20)	3.0e^−07^	5654133	5654384	GTTAAAACCCTCTAAAATCCCTATCAGGGATTGAAAC
*A. variabilis* ATCC 29413 (CRISPR9)	3.0e^−07^	2395670	2398703	GTTTCAATCCCTGATAGGGATTTTAGAGGGTTTTAAC
*A. variabilis* ATCC 29413 (CRISPR18)	1.0e^−06^	5227213	5229282	GTTTCTATTAACACAAATCCCTATCAGGGATTGAAAG
*Cyanothece* sp. strain PCC 7424 (CRISPR26)	4.0e^−06^	3575124	3575742	GTTACAATTAAAATGAATCCCTATTAGGGATTGAAAC
*A. variabilis* ATCC 29413 (CRISPR16)	4.0e^−06^	4821250	4823752	GTTGCAACACCACATAATCCCTATTAGGGATTGAAAC

The CRISPR array found in cyanophage N-1 is similar to the DR5 family of CRISPRs commonly found in cyanobacteria and is predicted to have the same characteristic hairpin structure (11) found in DR5 (type I-D) CRISPR repeats (data not shown) (51, 52). The CRISPR-Cas system is widespread among cyanobacteria, with 86 out of 126 sequenced genomes containing CRISPR-Cas systems and with multiple CRISPR arrays in many genomes (52). This includes *Nostoc* sp. strain PCC 7210 and *A. variabilis* ATCC 29413, which include 13 and 11 CRISPR arrays and 106 and 183 spacers, respectively (53). The sequence similarity of CRISPR repeats between cyanobacteria and N-1 suggests that the N-1 CRISPR was transferred from a cyanobacterium to an ancestor of N-1 during an infection, confirming that the CRISPR-Cas system has been exchanged by lateral gene transfer among microorganisms (12, 54).

Although no *cas* genes were identified in the N-1 genome, the N-1 CRISPR array was transcribed during infection (see Fig. S2 in the supplemental material). Possibly, N-1 contains unidentified genes encoding Cas proteins or host Cas proteins may be used for initiation. CRISPR loci can function without proximate *cas* genes (11, 54), and different CRISPR loci with similar repeats in the same genome can use the same set of Cas proteins. In *Nostoc* sp. strain PCC 7210, CRISPR8 is adjacent to a *cas* operon and is similar to the repeats in the N-1 CRISPR (Fig. 5D). Nothing is known about the expression of the CRISPR-Cas system in *Nostoc*, but in another type I CRISPR-Cas system in *Escherichia coli*, Cas proteins are continuously transcribed (55). This provides a mechanism for expression of the N-1 CRISPR array, even in the absence of virus-encoded Cas proteins.

The previous isolation of phages infecting *V. cholerae* that encode a CRISPR-Cas system demonstrated that the system defeats an inhibitory chromosomal island of the bacterial host (56). CRISPR arrays were also recently identified in prophages of *Clostridium difficile* (57). The prophage-carried CRISPR arrays were generally located in the structure/morphogenesis module and were similar to CRISPR arrays in the host, *C. difficile*. The prophage-carried CRISPR arrays contained spacers that match other *C. difficile* phage sequences and hence likely confer resistance to other phages that can infect the host. In the case of the N-1 CRISPR, the origin of the spacers is unknown, which makes it impossible to identify the target of the array. However, the CRISPR array in cyanophage N-1 may act as a mechanism against coinfection and offer a fitness advantage to both the host and the virus by preventing lysis by a competing phage.

The ability of viruses to contain and likely transfer CRISPRs benefits both the host and the carrier virus by introducing new spacers that protect the host from a wider spectrum of viruses that are potential competitors of the carrier virus. Expression of the viral CRISPR may be a particular selective advantage of filamentous cyanobacteria, because molecules are believed to be exchanged through nonspecific junctions that connect the cytoplasm of adjacent cells (58). Viral particles likely cannot pass through these channels, while small molecules such as CRISPR RNA (crRNA) probably can. This could allow cells adjacent to those infected by N-1 to acquire CRISPR sequences that would confer immunity to infection by other competing viruses.

The presence of a CRISPR array in cyanophage N-1 implies that phages can acquire CRISPRs and suggests that they can also transfer them among host cells. As the spacers likely originate from other phages, transferring the array among hosts has the potential to confer host immunity to competing phages.

### Conclusion.

Cyanophages A-1 and N-1, which infect *Nostoc* sp. strain PCC 7120, belong to a previously unrecognized evolutionary lineage of tailed phages. Most of their predicted protein-coding genes have no obvious similarity to sequences in databases, and those that do are generally most similar to genes found in filamentous cyanobacteria. Also, the closest phylogenetic relative of the DNA polymerases in A-1 and N-1 is found in a giant plasmid in the host. Moreover, the presence of a CRISPR array in N-1 suggests that cyanophages may exchange CRISPR elements among cyanobacteria. Together, these findings indicate the important role that lateral gene transfer has played in forging the evolutionary trajectory of this previously unrecognized evolutionary lineage of phages.

## MATERIALS AND METHODS

### Cyanophage isolation, purification, DNA preparation, and genome sequencing.

Cyanophages A-1and N-1 (ATCC 27893-B16 and ATCC 27893-B15, respectively) are tailed phages belonging to the family *Myoviridae* that infect *Nostoc* sp. strain PCC 7120 (ATCC 27893). Cyanophages were amplified on *Nostoc* sp. strain PCC 7120 grown in 800-ml batch cultures in 1-liter Erlenmeyer flasks containing BG-11 medium (59) under constant illumination (33 µmol of photons m^−2^ s^−1^, photosynthetically active radiation) at 26°C with constant shaking at 75 rpm. Exponentially growing cultures were infected with either A-1 or N-1 at a multiplicity of infection (MOI) of about 1 and left for 4 to 7 days until transparent, indicating lysis. To prevent phage binding to the filter, sodium chloride was added to the lysate at a final concentration of 0.5 M and the cultures were incubated at 4°C for 1 h before filtration through a 1.2-µm-pore-size GC50 glass fiber filter (Advantec MFS, Dublin, CA) and twice through GVWP 0.22-µm-pore-size polyvinylidene low-protein-binding filters (Millipore, Bedford, MA). Subsequently, the viral particles were concentrated by polyethylene glycol (PEG) precipitation (60). Briefly, the filtered lysate was centrifuged at 10,000 × *g* for 10 min in a Sorvall RC-5C centrifuge (GSA rotor, 4°C) to remove cellular debris. PEG 6000 was added to the supernatant to a 10% final concentration, and the mixture was incubated overnight at 4°C with constant shaking. The PEG solution was then centrifuged at 16,000 × *g* for 20 min (GSA rotor, 4°C), the supernatant was removed, and the pelleted viruses were resuspended in 200 µl of BG-11 medium.

DNA was extracted by treating the resuspended pellet with DNase 1 and RNase A to remove free nucleic acids with the QIAamp MinElute Virus Spin kit (Qiagen, Mississauga, ON, Canada) in accordance with the manufacturer’s instructions. The DNA was sequenced by 454 GS FLX titanium pyrosequencing at the Génome Québec Innovation Centre, McGill University (Montreal, Quebec, Canada). For each phage, >36,000 reads with an average length of ~350 bp were assembled into three contiguous sequences (contigs) with the GS *de novo* Assembler (Roche) and closed into a single circular contig by PCR. The sequencing coverage was approximately 179-fold for A-1 and 250-fold for N-1.

### Genome annotation.

ORFs were predicted with GeneMark (61) and GLIMMER (62). To create the final predictions, the ORF calls from the two programs were combined. When ORFs predicted by both programs differed in size, the longer of the two was kept. The final set of predicted ORFs was translated and assigned putative functions by comparison with known protein sequences found in the GenBank (nr), Acclame, and Procite databases with the BLASTp program. The ORFs were considered to be homologous to a protein-encoding gene if the E value was <10^−4^. Identification of tRNA genes was performed with tRNAscan-SE (63). Putative promoter motifs were identified with PHIRE (38) and the default parameters of 20-mer DNA sequences (S) with 4-bp degeneracy (*D* = 4). The motif was considered a putative promoter if it was found in the 150-bp region immediately upstream of the start codon of a predicted protein-coding gene. Sequence logos of the motifs were created with WebLogo by using the alignment of the sequences (64). Identification of rho-independent terminators was performed with FINDTERM (Softberry, Inc.). The default energy threshold was set to −16 kcal for the analysis as previously described (25).

### Phylogenetic analysis.

The ORFs with similarity to genes coding for dCTP deaminase, DNA Pol B, and the large terminase subunit were used for phylogenetic analysis. Inferred amino acid sequences for the dCTP deaminase and DNA Pol B were aligned in ClustalX with default parameters, while the large terminase gene (*terL*) was aligned by using the Promals web server (65, 66) with default parameters. Geneious v4.7 (67) was used to manually refine the alignment and construct neighbor-joining trees. Maximum-likelihood (ML) trees were constructed with the RAxML Web-Server rapid bootstrapping and ML search (100 replicates) (68) assuming the James-Taylor Thornton model of substitution with empirical base frequencies and estimating the proportion of invariable sites from the data.

### Gene comparison with other bacteriophages.

Predicted ORFs were compared to a database for T4-like phages infecting heterotrophic bacteria (10) and marine (16, 35) and freshwater (1) cyanobacteria (26). An ORF was considered shared if the E value was <10^−3^.

### Analysis of the CRISPR array in cyanophage N-1.

During the genomic analysis of N-1, a repeat DNA region was found and identified as a CRISPR array by CRISPRFinder (69). To show that this region was not a sequencing or assembly error, the CRISPR region was checked by PCR and sequencing. Two microliters of a 0.22-µm-filtered N-1 lysate was added to a 48-µl PCR mixture containing Platinum *Taq* DNA polymerase assay buffer (50 mM KCl, 20 mM Tris-HCl, pH 8.4), 10 mM MgCl_2_, 200 µM deoxynucleoside triphosphate, 0.25 µM each primer (sCRF [CAATTGGCAAAAGATTTAGCAGC] and CR3R [GGGGAGAGGTTTGGAGAGGGGT]), and 2.0 U of Platinum *Taq* DNA polymerase (Invitrogen, Carlsbad, CA). Negative controls contained all of the reagents but with sterile water as the template. PCR was carried out by denaturation at 94°C for 5 min; 35 cycles of denaturation at 94°C for 30 s, annealing at 57°C for 45 s, and extension at 72°C for 1 min; and a final extension at 72°C for 10 min (70). The amplification products were subjected to electrophoresis with 1.5% agarose–0.5× Tris-borate-EDT buffer (45 mM Tris-borate, 1 mM EDTA [pH 8.0]) at 100 V for 60 min. Gels were stained with GelGreen (Invitrogen) and visualized under UV illumination. The PCR amplicons were sequenced by Sanger sequencing.

Cyanophage N-1 DRs were compared with those from the CRISPR database with a BLASTN E value of <10^−5^. A sequence logo of the aligned consensus DRs from N-1 with the consensus of CRISPR 13 in *Nostoc* sp. strain PCC 7210 was created with WebLogo (64). The genomes of N-1 and its host were screened for *cas* genes by using the nr database. A neighbor-joining tree (Jukes-Cantor model) was created with the consensus DRs from the N-1 CRISPR and other cyanobacterial CRISPRs with Geneious (67) and edited with FigTree v1.3.1 (http://tree.bio.ed.ac.uk/software/figtree).

### RNA isolation and RT-PCR.

To confirm the transcription of the N1 CRISPR loci, the presence of precursor crRNA (pre-crRNA) was analyzed by reverse transcriptase PCR (RT-PCR). Total RNA was extracted from host cells infected with N-1. Two flasks, each containing 100 ml of an exponentially growing *Nostoc* sp. strain PCC 7210 culture, were infected with N-1 lysate, and 15 ml was collected at day 5. *Nostoc* cells were pelleted by centrifugation and resuspended in BG-11 medium. RNA was extracted with TRIzol reagent in accordance with the manufacturer’s protocol (Life Technologies). Briefly, 0.75 ml of TRIzol reagent was added to the resuspended pellets. The cells were lysed by being pipetted up and down several times. The samples were centrifuged at 12,000 × *g* for 10 min at 4°C (Beckman Coulter Allegra X-22R). The supernatant was then transferred to a new microcentrifuge tube and incubated for 5 min at room temperature to permit complete dissociation of the nucleoprotein complex. A 0.2-ml volume of chloroform was then added to the tube, and it was incubated for 3 min at room temperature. The sample was then centrifuged for 15 min as described above, and the resulting aqueous phase was collected and placed into a new microcentrifuge tube. A 0.5-ml volume of 100% isopropanol was added to the aqueous phase, which was incubated at room temperature for 10 min. The sample was centrifuged for 10 min at 4°C as described above, the supernatant was removed from the tube, and the pellet was washed with 1 ml of 75% ethanol. The RNA pellet was then resuspended in 50 µl of RNase-free water.

RT-PCR targeted the sequence between spacers 1 and 4 (~150 bp). First, an aliquot of the extracted RNA was treated with DNase I (Invitrogen) to remove DNA. The cDNA was generated with Superscript III RT (Invitrogen) by using random hexamers (50 ng/µl). Amplification was carried out in 25-µl PCR mixtures containing 10 ng of a cDNA template, 1 µM each primer, 1.5 mM MgCl_2_, 0.2 mM deoxynucleoside triphosphates, and 0.5 U of Platinum *Taq* DNA polymerase (Invitrogen). The PCR cycle parameters were a single denaturation step of 95°C for 5 min; 35 cycles of 95°C for 30 s, 57°C for 1 min, and 72°C for 3 min; and a final extension step of 72°C for 10 min.

## Nucleotide sequence accession numbers.

The nucleotide sequences of the dsDNA genomes of cyanophages A-1 and N-1 have been deposited in the GenBank database under accession no. KU234533 and KU234532, respectively.

## SUPPLEMENTAL MATERIAL

Figure S1  ML phylogenetic tree of dCTP deaminase protein sequences from viruses and bacteria. Bootstrap values of 90 to 100% (black circles) and 75 to 89% (gray circles) are shown at the nodes. Cyanophages N-1 and A-1 are denoted by asterisks. The scale bar shows the number of amino acid substitutions per site. Download Figure S1, TIF file, 1.4 MB

Figure S2  Transcription of the N-1 CRISPR into pre-crRNA in two *Nostoc* cultures infected with cyanophage N-1 (cultures A and B). The N1-CRISPR RT-PCR products were separated on a 1.5% agarose gel and stained with GelGreen. The lanes on the gel are as follows: M, Invitrogen 100-bp ladder; 1, PCR negative control; 2, culture A RNA control; 3, culture A cDNA; 4, culture B RNA control; 5, culture B cDNA; 6, RT negative control; 7, PCR positive control. Download Figure S2, EPS file, 2.1 MB

Table S1  Predicted ORFs of cyanophage A-1 (L) with homology to sequences in the nr database. Table S1, DOCX file, 0.1 MB

Table S2  Predicted ORFs of cyanophage N-1 with homology to sequences in the nr database. Table S2, DOCX file, 0.1 MB

Table S3  Spacer sequences in the CRISPR array. Table S3, DOCX file, 0.04 MB
