# Supplementary figures and images for: Viruses Infecting a Freshwater Filamentous Cyanobacterium (Nostoc sp.) Encode a Functional CRISPR Array and a Proteobacterial DNA Polymerase B
Source: mBio. 2016 Jun 14;7(3):e00667-16. doi: 10.1128/mBio.00667-16 (PMC4916379; doi:10.1128/mBio.00667-16)

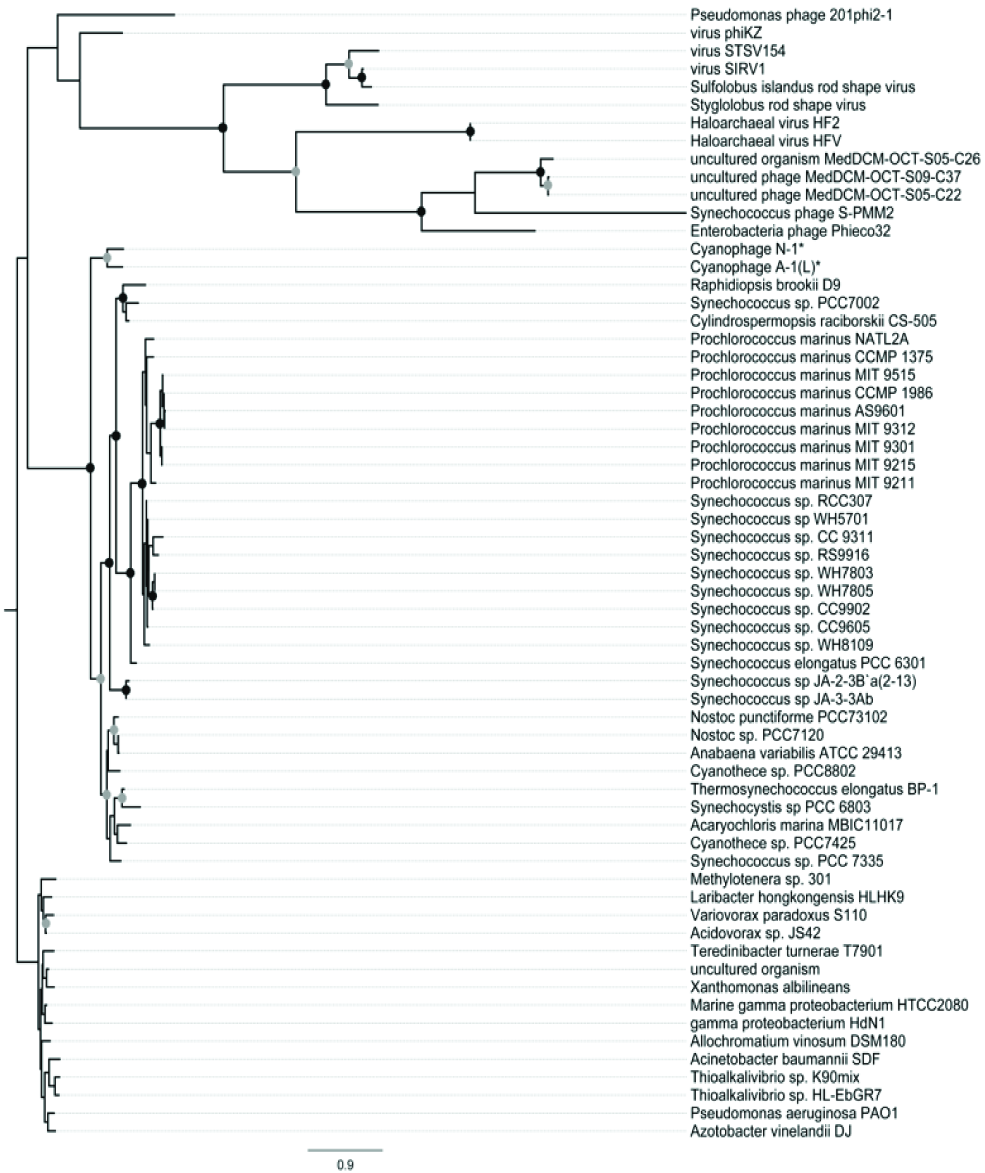

Supplement: Figure S1 — ML phylogenetic tree of dCTP deaminase protein sequences from viruses and bacteria. Bootstrap values of 90 to 100% (black circles) and 75 to 89% (gray circles) are shown at the nodes. Cyanophages N-1 and A-1 are denoted by asterisks. The scale bar shows the number of amino acid substitutions per site. Download [file mbo003162845sf1.tif]
