# Supplementary material for: Viruses Infecting a Freshwater Filamentous Cyanobacterium (Nostoc sp.) Encode a Functional CRISPR Array and a Proteobacterial DNA Polymerase B
Source: mBio. 2016 Jun 14;7(3):e00667-16. doi: 10.1128/mBio.00667-16 (PMC4916379; doi:10.1128/mBio.00667-16)
Supplement: Table S1 — Predicted ORFs of cyanophage A-1 (L) with homology to sequences in the nr database. [file mbo003162845st1.docx]

**Supplementary Table 1.**

| **ORFs** | **Length(bp)** | **Strand** | **Significant hit** | **Organism** | **e-value** | **%identity (shared aa)** |
| --- | --- | --- | --- | --- | --- | --- |
| 1 | 1359 | F | Terminase large subunit | Nostoc sp. PCC 7524  AFY48994.1 | 2.28e^-54^ | 30% (129) |
| 2 | 1605 | F | Hypothetical protein | Nostoc sp. PCC 7524 AFY48995.1 | 7.16e^-17^ | 21%(113) |
| 3 | 702 | F | Outer membrane protein (OmpH-like) |  |  |  |
| 4 | 1098 | F | putative Major capsid protein | Cyanophage AN-15 | 7.00e-^159^ | 77.6(288) |
| 6 | 327 | F | Hypothetical protein | Lactobacillus phagage KC5a | 3.94e^-03^ | 29.1%(25) |
| 9 | 1521 | F | Tail sheath protein | Nostoc sp. PCC 7524 AFY49006.1 | 1.14e^-69^ | 38% (137) |
| 10 | 504 | F | T4-like virus tail tube protein |  |  |  |
| 13 | 2070 | F | Phage-related tail | Burkholderia cenocepacia MC0-3 | 2.1e^-08^ | 38%(50) |
| 15 | 1182 | R | Zinc metallopeptidase M23-family/Phage late control gene D protein | Sagittula stellata E-37 | 2.11e^-08^ | 50.7%(38) |
| 16 | 1416 | R | Lysozyme-like domain,Rare lipoprotein A | Anabeana variabilis ATCC 29413 | 2.3e^-19^ | 57.7%(60) |
| 19 | 738 | R | Hypothetical protein | Nostoc sp PCC7524 (AFY49010.1) | 9.39e^-11^ | 29%(67) |
| 23 | 891 | R | Exonuclease RNase T and DNA polymerase | Cyanobacterium aponinum PCC10605 | 1.63e^-08^ | 27% (48) |
| 24 | 438 | R | Hypothetical protein | Geobacillus sp Y412MC10 | 5.6e^-15^ | 43.6%(44) |
| 25 | 405 | F | LuxR-family regulator protein, helix-turn-helix motif | Clostridium acetobutylicum ATCC 824 | 8.10e^-05^ | 42.6(54) |
| 26 | 813 | F | Phage-related baseplate assembly protein | Nostoc sp PCC7524 (AFY49015.1) | 1.46e^-32^ | 34%(91) |
| 32 | 738 | F | BaseplateJ phage tail | Nostoc sp PCC7524 (AFY49018.1) | 3.76e^-33^ | 41% (94) |
| 34 | 576 | F | Phage tail protein (tail_P2_I) | Nostoc sp PCC7524 (AFY49020.1) | 5.63e^-40^ | 59%(77) |
| 35 | 1389 | F | Hypothetical protein | Nostoc sp PCC7524 (AFY49021.1) | 4.83e^-57^ | 37%(157) |
| 36 | 1140 | F | Tail collar protein | Nostoc sp PCC7524 (AFY49022.1) | 2.1e^-52^ | 50% (149) |
| 39 | 2016 | R | DNA polymerase B | Cyanothece PCC7425 | 7.00e^-115^ | 40.4% (237) |
| 43 | 1428 | R | Type III restriction enzyme,DEAD-box helicase | Lactobacillus phage phiadh | 2.00e^-21^ | 25.8%(91) |
| 54 | 939 | R | putative ant AntA/AntB antirepressor | Leptolyngbya sp. PCC 7375 | 1.90e^-24^ | 45%(51) |
| 59 | 618 | R | DNA N-6-adenine-methyltransferase | Synechocystis sp. PCC 7509 | 1.00e^-06^ | 27.3%(44) |
| 66 | 243 | R | ASCH domain protein | Clostridium symbiosum ATCC 14940 | 9.87e^-16^ | 44%(35) |
| 68 | 564 | R | Hypothetical protein | Bacillus megaterium WSH-002 | 1.03e^-08^ | 27.7%(44) |
| 70 | 3066 | R | putative DNA primase | Streptococcus thermophilus CNRZ1066 | 7.73e^-03^ | 20.4%(80) |
| 72 | 1209 | F | Transposase | Nostoc sp. PCC7120 | 0 | 388(100%) |
| 73 | 639 | F | Thymidylate kinase | Raphidopsis brooki D9 | 1.5e^-31^ | 38.6% (78) |
| 75 | 417 | F | Hypothetical protein | Calothrix sp. PCC 7103 | 2.59e-^48^ | 58% (98) |
| 77 | 1044 | F | DNA-cytosine methyltransferase | Thrichodesmium erythraeum IMS101 | 3.0e^-62^ | 37.8%(140) |
| 83 | 507 | R | Hypothetical protein | Roseophage DSS3p2 | 5.00e^-29^ | 43.2%(64) |
| 84 | 726 | R | Hypothetical protein | Acidovorax avenae subsp. citrulli AAC00- | 1.00e^-07^ | 29.6%(32) |
| 86 | 600 | F | dCTP deaminase/dUTPase superfamily | Cyanothece PCC7425 | 2.00e^-76^ | 67.3%(134) |
| 89 | 888 | F | DNA methylase N-4/N-6 domain protein | Arthrospira maxima | 1.00e^-65^ | 49.4%(133) |
| 90 | 432 | F | endodeoxyribonuclease RusA | Cyanothece PCC7425 | 8.00e^-04^ | 28.4%(29) |
| 92 | 780 | R | Thymidylate synthase complementing protein | Chlorobium phaeobacteroides BSI | 4.38e^-39^ | 38.4 %(86) |
