# Supplementary material for: Viruses Infecting a Freshwater Filamentous Cyanobacterium (Nostoc sp.) Encode a Functional CRISPR Array and a Proteobacterial DNA Polymerase B
Source: mBio. 2016 Jun 14;7(3):e00667-16. doi: 10.1128/mBio.00667-16 (PMC4916379; doi:10.1128/mBio.00667-16)
Supplement: Table S2 — Predicted ORFs of cyanophage N-1 with homology to sequences in the nr database. [file mbo003162845st2.docx]

**Supplementary Table 2.**

| **ORFs** | **Length**  **(bp)** | **Strand** | **Significant hit** | **Organism** | **e-value** | **%identity (shared aa)** |
| --- | --- | --- | --- | --- | --- | --- |
| 1 | 1359 | F | phage terminase, large subunit | *Nostoc* sp PCC7524 | 2.32e^-55^ | 31%(134) |
| 2 | 1602 | F | Hypothetical protein | *Nostoc* sp PCC7524  (AFY48995) | 3.55e^-18^ | 22%(106) |
| 3 | 696 | F | Outer membrane protein (OmpH-like) |  |  |  |
| 4 | 1089 | F | putative major capsid protein | Cyanophage AN-15 | 1.94e^-134^ | 72%(254) |
| 6 | 330 | F | Hypothetical protein | *Nostoc* sp PCC7524  (AFY49001) | 9.62e^-06^ | 30%(30) |
| 9 | 1521 | F | Tail sheath protein | *Nostoc* sp PCC7524  (AFY49006) | 3.74e^-74^ | 39%(138) |
| 10 | 498 | F | T4-like virus tail tube |  |  |  |
| 13 | 2079 | F | phage-related tail trans could also be phage tail tape measure protein | Vibrio phage VHML | 1.22e^-7^ | 34%(42) |
| 15 | 2694 | R | Lysozyme-like domain, rare lipoprotein A (RlpA)-like double psi beta barrel | *Nostoc* sp PCC7524  (AFY49014) | 1.48e^-37^ | 28 % (137) |
| 18 | 849 | R | Hypothetical protein | *Nostoc* sp PCC7524  (AFY49010) | 9.82e^-10^ | 27.9%(51) |
| 19 | 918 | F | Exonuclease RNase T and DNA polymerase III | *Thauera* sp MZIT | 4.14e^-04^ | 28.4%(38) |
| 24 | 387 | F | LuxR family regulatory protein, helix-turn-helif motif | *Streptomyces* *albus* | 3.15e^-06^ | 40.3% (25) |
| 25 | 813 | F | gp5 baseplate hub subunit and tail lysozyme | Acinetobacter phage Ac42 | 4.24e^-07^ | 28.6%(30) |
| 29 | 330 | F | Lysosyme | *Nostoc* sp PCC7524  (AFY49017) | 2.51e^-03^ | 24%(32) |
| 30 | 1167 | F | Baseplate J phage tail protein | *Nostoc* sp PCC7524  (AFY49018) | 4.18 e^-68^ | 43%(144) |
| 31 | 576 | F | Phage tail protein | *Nostoc* sp. PCC 7524  (AFY49020) | 2.32e^+00^ | 26.3% (45) |
| 32 | 1395 | F | Phage tail fiber protein | *Nostoc* sp. PCC 7524  (AFY49021) | 2.97e^-64^ | 37%(172) |
| 33 | 1155 | F | Tail collar protein | *Nostoc* sp. PCC 7524  (AFY49022) | 1.62e^-47^ | 33%(137) |
| 34 | 1896 | R | DNA polymerase B | *Cyanothece* sp. PCC 7424 | 7.0e-^118^ | 41.5%(243) |
| 38 | 1410 | R | Putative DEAH-family helicase | Lactobacillus phage phiadh | 2.61e^-31^ | 28.1%(123) |
| 48 | 600 | R | Hypothetical protein | *Nostoc* *punctiforme* PCC 73102 | 1.10e^-06^ | 21.4%(40) |
| 52 | 633 | R | C-5 cytosine-specific DNA methylase | *Nostoc* *punctiforme* PCC 73102 | 1.81e^-05^ | 36.2%(34) |
| 56 | 240 | R | ASCH domain protein | *Clostridium* phage phiMMP02 | 4.06e^-12^ | 42.3%(33) |
| 58 | 588 | R | Hypothetical protein | *Bacillus* phage SPBc2 | 7.44^e-05^ | 22.6%(35) |
| 61 | 2739 | R | putative DNA primase | *Listeria* *welshimeri* *serovar* 6b str. SLCC5334 | 6.16e^-04^ | 20.2% (68) |
| 62 | 627 | F | Thymidylate kinase | *Lyngbya* PCC8106 | 2.45e^-23^ | 36%(74) |
| 68 | 1047 | F | DNA-cytosine methyltransferase | *Anabaena* *variabilis* ATCC 29413] | 1.02e^-56^ | 33.2(127) |
| 75 | 579 | R | Hypothetical protein | *Thermoanaerobacter* *italicus* Ab9 | 8.67e^-03^ | 32.7%(33) |
| 76 | 507 | R | Hypothetical protein | *Calothrix* sp. PCC 7103 | 1.73e-^47^ | 56%(94) |
| 77 | 726 | R | Hypothetical protein | *Calothrix* sp. PCC 7103 | 4.94e^-06^ | 30.4%(78) |
| 79 | 585 | F | dCTP deaminase | *Synechococcus* sp. PCC 7335 | 1.45e^-69^ | 65.5%(131) |
| 82 | 411 | F | endodeoxyribonuclease RusA | *Desulfotomaculum* *reducens* MI-1] | 3.99e^-01^ | 37.7%(29) |
| 85 | 741 | R | Thymidylate synthase complementing protein/FAD-dependent thymidylate synthase | *Chlorobaculum* *parvum* NCIB 8327 | 8.35e^-33^ | 39.1%(86) |
