# Supplementary material for: Viruses Infecting a Freshwater Filamentous Cyanobacterium (Nostoc sp.) Encode a Functional CRISPR Array and a Proteobacterial DNA Polymerase B
Source: mBio. 2016 Jun 14;7(3):e00667-16. doi: 10.1128/mBio.00667-16 (PMC4916379; doi:10.1128/mBio.00667-16)
Supplement: Table S3 — Spacer sequences in the CRISPR array. [file mbo003162845st3.docx]

**Supplementary Table 3.** Spacer sequences in the CRISPR array

| **Spacer** | **Length** | **%GC content** | **Sequence** |
| --- | --- | --- | --- |
| 1 | 34 | 32.4 | CAATTGGCAAAAGATTTAGCAGCTTTTTTGATC |
| 2 | 29 | 24.1 | TGTAAAGTACTCTTCACAAATTCAAAACAAAAATAC |
| 3 | 33 | 54.5 | CCAAAGTACCATCGGCACTTCTTGTCCACCGGA |
| 4 | 37 | 35.1 | TCTCATAAAAGATTTTCGTCGCAATGCAACAAAAGCT |
